# Bite Me: Bark Stripping Showed Negligible Effect on Volume Growth of Norway Spruce in Latvia

**DOI:** 10.3390/plants13152014

**Published:** 2024-07-23

**Authors:** Agnese Anta Liepiņa, Sabīne Ieviņa, Endijs Bāders, Gundega Done, Roberts Matisons, Ieva Jaunslaviete, Beate Bērziņa, Āris Jansons

**Affiliations:** Latvian State Forest Research Institute Silava, Rīgas Str. 111, LV-2169 Salaspils, Latvia

**Keywords:** linear models, ungulates, cervids, growth trends, *Picea abies* (L.) Karst

## Abstract

Over the past few decades, increasing populations of cervid species in the Baltic region have reduced the quality and vitality of cultivated Norway spruce (*Picea abies* (L.) Karst.) stands. This study evaluated the effect of bark stripping on the volume growth of spruce trees in Latvia. Data collection took place in two forest stands. In each stand, 20 Norway spruce trees were sampled, 10 with visible bark damage scars and 10 control trees. Stem discs were collected from control trees at specified heights (0 m, 0.5 m, 1 m, 1.3 m, and 2 m, and then at one-metre intervals up to the top) and from damaged trees at additional specific points relative to the damage. Each disc was sanded and scanned; tree ring widths were measured in 16 radial directions using WinDendro 2012a software. Annual volume growth reconstruction was performed for each tree. Changes in relative volume growth were analysed in interaction with scar parameters, tree type (damaged/control), and pre-damage volume using linear regression models. The significance of parameter interactions was assessed using analysis of variance (ANOVA). Pairwise comparisons of estimated marginal means (EMMs) were conducted using Tukey’s HSD post hoc test. No significant effect of bark stripping on the total stem volume increment was detected. However, the length of bark stripping scars had a significant impact on relative volume growth in the lower parts of the stems. These findings underscore the importance of further research examining a broader spectrum of cervid damage intensity and the effects of repeated damage on tree survival and growth.

## 1. Introduction

In forests of the Northern hemisphere, cervid species influence the growth and survival of various tree species, thus maintaining the heterogeneity of forest ecosystems [1,2,3]. However, browsing can also negatively affect forest and agricultural production [4,5,6]. Over recent decades, populations of large herbivores, particularly those in the *Cervidae* family, have increased across extensive regions of central and northern Europe [7,8], thus influencing natural and cultivated vegetation.

Cervids alter forest structure and species composition through browsing, fraying trees with antlers, and stripping bark [9]. These activities can result in the death of overstory trees, and when combined with the browsing of regeneration, they can alter forest structures or lead to local deforestation [10,11,12]. Bark stripping by deer can cause severe damage to production trees, leading to economic losses from reduced growth, fungal infestation, stem deformation, and wood decay [13,14]. Bark stripping during spring can cause extensive damage to tree water conductivity since the nutritional balance and increased starch content of the bark become increasingly appealing for cervids [15]. Fungal infection and limited water uptake further increase stand sensitivity to wind and snow damage [16,17,18,19,20,21]. However, cervids prefer certain tree species, with Norway spruce (*Picea abies* (L.) Karst.), European ash (*Fraxinus excelsior* L.), Sweet chestnut (*Castanea sativa* (Mill.)), and *Sorbus* spp. being the most affected in Europe [9].

Even-aged monocultures of Norway spruce with high stand density have been strongly affected by bark stripping [9,22,23], likely due to limited alternative foraging possibilities on the ground within these stands [24,25]. In central Europe, bark stripping reduces the annual growth of spruce, sometimes as a systematic response, occurring in both damaged and undamaged portions of trees [26]. Bark stripping greatly damages saplings and trees by obstructing water conductivity and increasing spruce’s susceptibility to illnesses [27,28]. Since bark stripping occurs at the lower parts of the stem (usually up to 2 m), most pathogenic infections and hence most of the wood decay affect the thickest and therefore most valuable part of the stem [13,29,30]. The associated reduction in usable timber quantity and quality leads to a decrease in timber yields [31].

In Latvia, during the past decade the intensity of bark stripping and the number of affected stands have been increasing [32], reducing the quality and vitality of cultivated spruce stands; however, little is known about how these damages impact the volume growth of spruce in the Baltic region. The two main species causing bark-stripping damage to spruce stands in Latvia are red deer *Cervus elaphus* (L.) and moose *Alces alces* (L.). This study aimed to evaluate the effect of bark stripping damage caused by red deer on the volume growth of spruce trees. We hypothesised that the relative volume growth of damaged spruce trees is going to decrease in the post-damage period, as the trees will likely divert resources towards defence and recovery efforts rather than growth.

## 2. Materials and Methods

This study was conducted at a hemiboreal forest site in Latvia (57°14′ N; 22°40′ E), where the climate is temperate, with the mean temperature ranging from −1.6 ± 2.3 °C in January to 17.6 ± 1.6 °C in July [33]. The mean annual temperature was 7.4 ± 0.7 °C. The mean precipitation reached 682.5 ± 81.6 mm per year.

Two stands of maturing Norway spruce were sampled. The site conditions of both stands were characterised by mesotrophic, well-drained mineral soils (*Oxalidosa*). Stand A (57°14′39.658″ N; 22°40′38.819″ E) had an area of 0.47 hectares, the age of the stand at the sampling moment (in 2020) was ~31 years, and the canopy layer consisted purely of Norway spruce. Stand B (57°14′31.755″ N; 22°41′57.852″ E) had an area of 1 hectare; the age of the trees was ~40 years. Stand B was dominated by spruce with ~10% admixture of birch (*Betula pendula* Roth).

To assess growth changes in Norway spruce, 20 trees were cut in each stand—10 with visible bark damage scars from cervids and 10 control trees without damage. Trees for which the diameter at breast height (DBH) was ≥10 cm and, in the case of the damaged trees, for which the width of the bark stripping scar was ≥5 cm were sampled (Figure S1).

Stem discs were obtained from all sampled trees; for control trees, the discs were cut at heights of 0 (at the root collar), 0.5, 1, 1.3, and 2 m, and then at intervals of one metre until the very top of the tree. In the case of the damaged trees, stem discs were obtained at the root collar (0 m), 0.5 m above the root collar, 0.5 m below the damage, directly below the damage, at 1 m, 1.3 m, the middle of the damage, directly above the damage, 0.5 m above the damage, and 1 m above the damage (Figure S2). Furthermore, the discs were cut at one-metre intervals up to the treetop.

Each disc was sanded, and scanned images (resolution of 600 dpi) of the upper part of the discs were obtained using an EPSON Expression 12000XL and the 16-bit grayscale mode. The annual tree ring widths (TRW) were measured using the WinDendro software (Regent Instruments Inc., Québec, QC, Canada) in 16 radial directions (calibrated at the pith).

To evaluate whether bark stripping had induced changes in spruce volume increment, the reconstruction of volume growth was performed individually for each tree on an annual basis. Using the TRW measurements, the mean annual TRW for each disc was calculated. Furthermore, the basal area increment (BAI) of the lower and upper part (stem disc) of each section of the stem was calculated (starting from the pit). Then, utilising the height of the stem sections (length between the cut stem discs) and the mean BAI (from the upper and lower part of each stem section), the annual volume increment of each separate stem section was calculated, assuming that the height increment had been similar across the years. Furthermore, by combining the annual volume increments of stem sections, the annual volume increment of an entire tree was obtained. The cumulative volume increment was calculated to analyse the spruce growth dynamics.

To assess the changes in relative additional volume increment after the year of bark stripping damages (representing the species’ resilience), a ratio of the mean stem volume increments five years prior and five years after the damage was calculated. In the case of control trees, the ratio was calculated similarly, using the same years (the year with the highest damage frequency for each stand). Furthermore, the total stem volume of each tree was divided into two sections: below two metres of height, where the damage scars were observed, and above the two-metre mark, to evaluate whether the damage had indirectly affected the upper part of the tree. Changes in relative volume growth in interaction with scar parameters, tree type (damaged/control), and volume before the damages were analysed using linear regression models. Analysis of variance (ANOVA) was used to assess the significance of parameter interactions. Additionally, the estimated marginal mean (EMM) values were calculated and compared using Tukey’s HSD post hoc test for pairwise comparisons of the factor interactions, determining the changes in additional relative volume growth of the spruce. Data were analysed using R software v. 4.4.1 [34].

## 3. Results

The years of damage varied between stands and individual trees; the year with the highest frequency of damaged trees for stand A was 2014, and for stand B the damage occurred earlier—mostly in 2006 and 2007. The age of the trees in stand A at the time when the bark stripping occurred ranged from 31 to 33 years. In stand B, the age of the trees at the year of damage ranged from 24 to 25 years, except for two trees that were damaged later in 2017 and 2018 (34 and 35 years old at the time). The mean scar height for trees ranged from 0.86 ± 0.10 m in stand B to 0.99 ± 0.03 m in stand A. The scanned images revealed that cervids had most often damaged the secondary phloem, thus disrupting the activity or causing the death of the vascular cambium within the stripped part of the stem (Figures S2 and S3).

The dimensions of the sampled spruce trees were similar amongst both stands. The mean DBH (±standard error) for the younger stand A was 18.2 ± 1.1 cm, ranging from 10.8 to 26.1 cm. The mean DBH for damaged and control trees was comparable—18.1 and 18.2 cm, respectively. For the older stand B, the mean diameter ranged from 16.4 ± 0.9 cm for control trees to 20.7 ± 0.8 cm for damaged trees. The mean height in stand A varied from 18.0 ± 1.49 to 18.3 ± 1.64 m for damaged and control trees, respectively. In stand B, the trees were slightly higher; the mean height of damaged trees was 22.4 ± 1.4 m and for control trees 21.2 ± 1.6 m.

**Figure 1:**
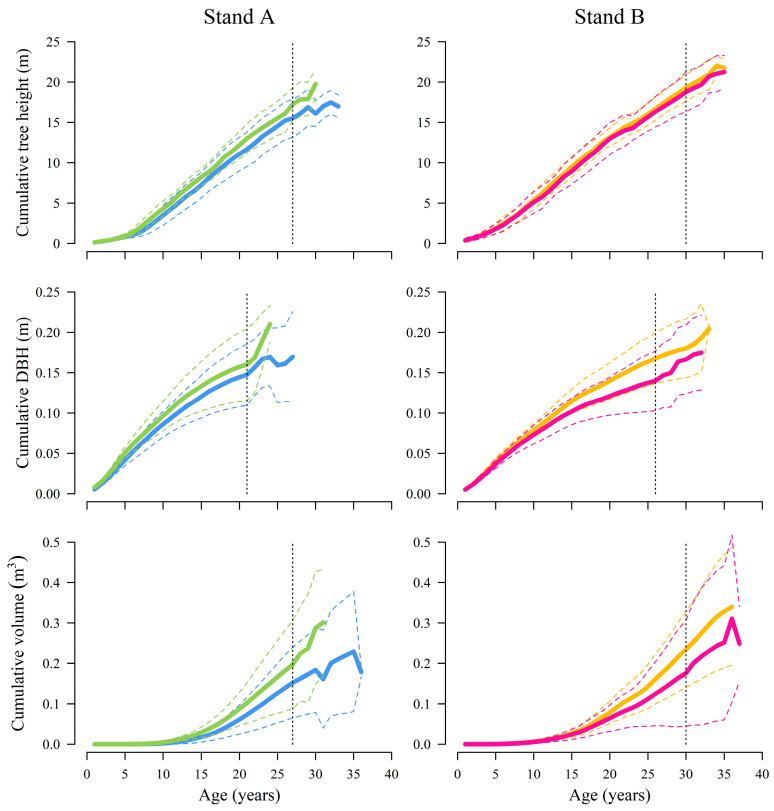
The mean observed height, DBH (without bark), and stem volume growth rate for damaged and control trees in both studied stands. For stand **A**, the mean (bold line) ± standard deviation (dashed lines) values for damaged trees are shown in blue, and for control trees in green. For stand **B**, the damaged tree growth is displayed in orange, and the growth of control trees is in pink. The vertical dashed line marks the age for which the mean growth values were calculated using data from all trees (tree age differed).

The height growth rate of damaged and control trees occurred similarly in stand A; however, the damaged trees showed a slightly slower growth rate than the control trees (Figure 1, stand A). Furthermore, comparable growth trends are visible in the case of DBH and stem volume growth, meaning that the control trees developed slightly greater dimensions faster than the damaged trees. Minor differences in the mean growth speed are detectable early, during the first ten years of growth, meaning that the dimensions of sampled trees already differed slightly before cervid damages.

In the case of stand B, the mean height growth rate of the sampled trees was substantially similar (damaged and control); however, due to slight differences in DBH growth rate, small differences in stem volume growth rate amongst the trees were detected (Figure 1, stand B). Up until the age of 15 years, the mean DBH growth of damaged and control trees occurred evenly; however, in later years the damaged trees showed slightly increased mean DBH and mean stem volume growth. In addition, after reaching the age of 25 years, the control trees displayed greater stem volume growth variation than the damaged trees. Similarly, the differences in mean growth trends that were described mostly occurred before the year of cervid damages.

The linear models demonstrated moderately good performance, explaining approximately 66.71% of the variance in relative volume growth for full stem volume and 68.32% below the 2 m stem sections; however, slightly less variance (62.07%) was explained in the case of the upper parts of the stems (above 2 m). The analysis of variance (ANOVA) revealed that the relative volume increment for the five years post damage year differed significantly amongst the stands (*p* < 0.001) (Table 1). However, no significant differences in relative volume growth were detected between the damaged and control trees (*p* ≥ 0.3). For the lower part of the stem (below 2 m), statistically significant differences in relative volume growth were observed based on the length of the bark stripping scars (*p* = 0.02); however, in the case of full stem volume and the upper section of the tree, the differences were marginally significant (*p* ≥ 0.06–0.09).

Across all parts of the tree (upper, lower, and full stem), control trees consistently show higher relative volume growth than damaged trees (Table 2). In forest stand A, regardless of whether the trees were damaged, the mean relative volume growth was greater than in stand B. This suggests that division by stands affects growth, and this effect is more pronounced above two metres compared to the lower part of the spruce stems. The differences between damaged and control trees within each stand category are less distinct, as indicated by the overlapping group letters in some cases, implying that while the type of the tree (damaged or controlled) may influence growth, its effect is not as strong or consistent as that of the stand that trees grow in.

## 4. Discussion

Cervid-induced damages have been shown to negatively affect forest stands, in some cases even leading to the death of overstory trees [4,6,7]. However, in the case of the spruce trees studied herein, no signs of tree mortality were observed before sampling. The observed damages likely did not exceed the severity of 65% circumference damage, which has been found to cause tree death [27]. Furthermore, bark stripping by cervids can lead to significant economic losses due to reduced growth, fungal infestations, stem deformation, and wood decay [13,14]. In contrast, our results revealed significant variability in relative volume increment amongst the stands (*p* < 0.001). However, no significant differences in relative volume growth between damaged and control trees were observed (Table 1; *p* > 0.3), thus implying the resilience of Norway spruce and contradicting the initial hypothesis.

Furthermore, bark stripping can induce a systematic response of diameter increment reduction for spruce, affecting the stems’ damaged and healthy parts [26]. In our case, the relative volume growth was greater in the upper parts of the stems compared to the lower parts (Table 2); significant differences were observed in the lower part of the stem based on the length of bark stripping scars (*p* = 0.02). This implies that the scars in the lower part of the trunk did not significantly affect the growth of the healthy parts of the trees. In addition, bark stripping during spring can lead to more extensive damage [13,30]. Since bark stripping did not cause significant differences in relative volume growth, we speculate that the damages most likely occurred during the winter season, when bark detaching is harder [29] and trees are less sensitive to damages in water conductivity.

In conclusion, bark stripping by cervids had a negligible effect on the short-term volume growth of Norway spruce in Latvia, implying the species’ resilience. The vertical dimensions of scars significantly affected volume growth only within the lower part of the stems, indicating that the immediate physical damage does not significantly hinder overall tree development. However, these findings highlight the need for further research into long-term impacts and potential secondary effects, such as illness susceptibility and altered wood quality. Additionally, examining a broader spectrum of damage intensity and the effects of repeated damage, considering local and stand-specific factors, will be crucial to better understand and manage the impacts of bark stripping on tree growth and survival.

## Figures and Tables

**Table 1:** Statistics of the linear regression model: F-value and, in the brackets, the corresponding *p*-value (*p*-values < 0.05 are shown in bold). Response variable—the mean annual relative volume increment of the five years after the year of Cervid damage. Predictor variables: tree type—control or damaged spruce trees; stand—forest stand (A or B); initial volume—the tree volume before the damage; scar width—the width of the scar at the widest point (horizontal); scar length (vertical). The parameter influence was assessed for the full stem volume and sections of stem volume above and below 2 m of height.

	Full Stem Volume	Stem Volume below 2 m	Stem Volume above 2 m
Tree type	0.47 (0.50)	1.0967 (0.30)	0.31 (0.59)
Stand	**57.25 (<0.001)**	**59.18 (<0.001)**	**45.49 (<0.001)**
Initial volume	3.03 (0.09)	1.88 (0.18)	1.80 (0.19)
Tree type × Initial volume	0.01 (0.92)	0.59 (0.45)	0.09 (0.76)
Scar width	0.72 (0.40)	0.06 (0.80)	1.02 (0.32)
Scar length	3.69 (0.06)	**6.70 (0.02)**	3.06 (0.09)

**Table 2:** Estimated marginal means (EMMs) of the five-year mean annual relative volume increment of spruce trees for each combination of tree type (control, damaged) and stands, including the ±95% confidence interval. The value comparison is displayed separately for each of the tree sections (horizontally)—for full stem volume, and tree stem volume above (stem above 2 m), and below (stem below 2 m) the damage. The same lowercase letters indicate a lack of significant differences (*p* > 0.05) between the pairwise comparisons of estimated marginal means. The comparisons are made for each variable independently.

	Stand A	Stand B
***Full stem volume***		
Control	0.778 ± 0.313 ab	1.538 ± 0.303 c
Damaged	0.621 ± 0.369 a	1.360 ± 0.291 bc
***Below 2 m***		
Control	0.596 ± 0.254 ab	1.169 ± 0.250 c
Damaged	0.271 ± 0.302 a	0.967 ± 0.238 bc
***Above 2 m***		
Control	0.837 ± 0.361 ac	1.639 ± 0.350 bd
Damaged	0.735 ± 0.426 ab	1.467 ± 0.337 cd

## Data Availability

Dataset available on request from the authors.

## Supplementary Materials

The following supporting information can be downloaded at: https://www.mdpi.com/article/10.3390/plants13152014/s1, Figure S1: Markings of sampling heights for stem discs of damaged spruce tree. Figure S2: A bark-stripping scar on the stem of a damaged spruce tree. Figure S3: A scanned image of the cross-section of a damaged spruce tree.
