# Supplementary figures and images for: Bite Me: Bark Stripping Showed Negligible Effect on Volume Growth of Norway Spruce in Latvia
Source: Plants (Basel). 2024 Jul 23;13(15):2014. doi: 10.3390/plants13152014 (PMC11313968; doi:10.3390/plants13152014)

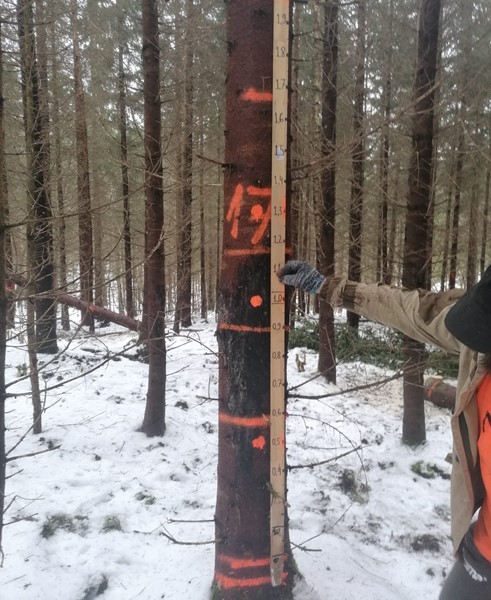

Supplement: Supplementary file 1 [file plants-13-02014-s001.zip › Figure_S1.jpg]

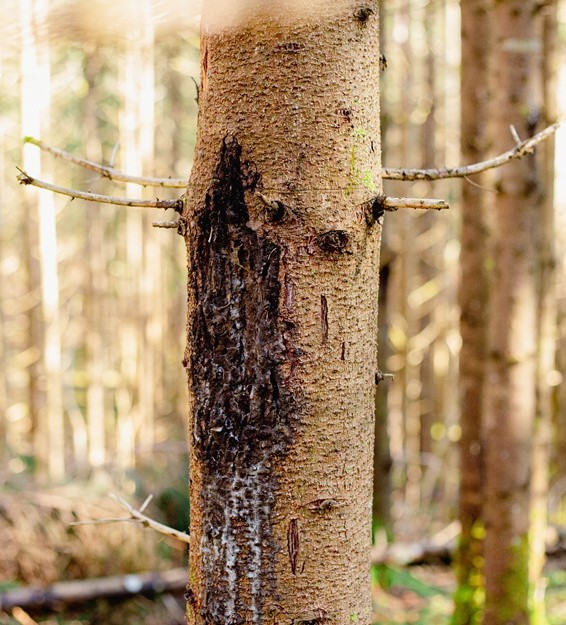

Supplement: Supplementary file 1 [file plants-13-02014-s001.zip › Figure_S2.jpg]

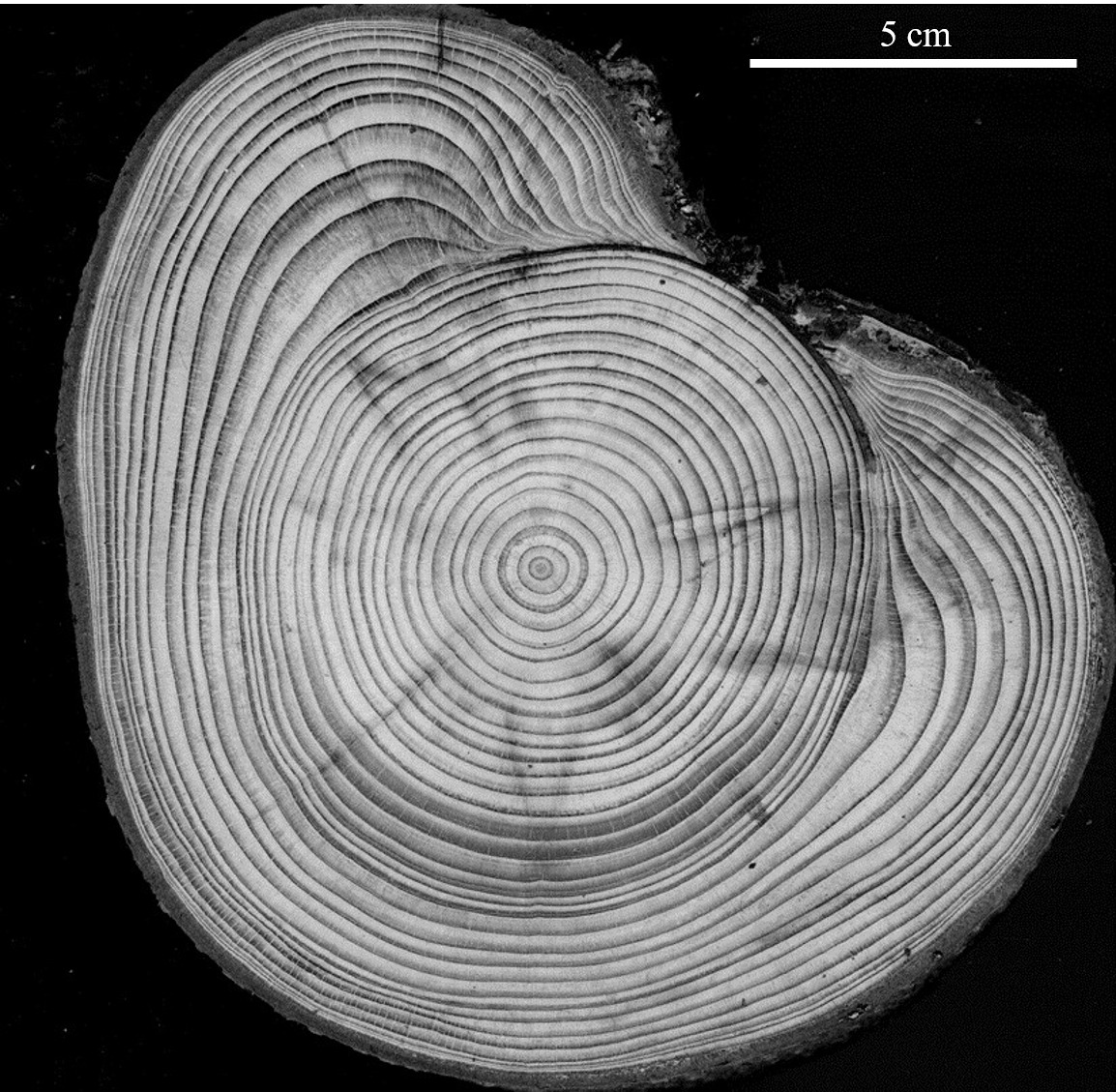

Supplement: Supplementary file 1 [file plants-13-02014-s001.zip › Figure_S3.png]
